# Factors and Reasons Associated With Appointment Non-attendance in Hospitals: A Narrative Review

**DOI:** 10.7759/cureus.58594

**Published:** 2024-04-19

**Authors:** Majed Alturbag

**Affiliations:** 1 School of Nursing & Midwifery, University of Dublin, Trinity College, Dublin, IRL

**Keywords:** patient adherence, hospital-specific reasons, patient-centred reasons, no-shows, missed appointments, non-attendance appointments

## Abstract

Non-attendance at hospital appointments is an extremely prevalent issue impacting healthcare systems on a daily basis. This phenomenon adversely affects patient health and healthcare providers, leading to delays in diagnosis and treatment, inefficient resource utilization, and increased healthcare expenses. The detrimental impact of non-attendance is not limited to patients who miss appointments, the knock-on effects of extended waiting times and reduced appointment availability are felt throughout healthcare systems. The purpose of this narrative review is to explore the factors underlying appointment non-attendance in hospital settings, to improve healthcare delivery and patient adherence. An extensive review of the existing global literature was conducted. Quantitative studies that explored the relationship between appointment non-attendance and patient characteristics, such as age, gender, marital status, education level, distance from the hospital, and source of referral, were included. Younger patients, males, individuals with lower levels of education, and those living farther from hospitals were more likely to miss appointments. Marital status was significant, with married patients showing better attendance, as was referral source, with general practitioner referrals associated with higher non-attendance. Qualitative studies identifying both patient-centered and hospital-specific reasons, such as forgetfulness, appointment time, protracted waiting times, patient-physician relationship, and patients’ knowledge and perception of their health condition, were also included in the review. Lack of appointment reminders, difficulties in managing appointments, and inadequate patient-physician communication were significant hospital-specific reasons given for non-attendance. Patients' lack of awareness regarding the importance of attending appointments and limited understanding of their health conditions were also identified as patient-centered contributors. Non-attendance at hospital appointments is a multifaceted issue influenced by a range of socioeconomic, personal, and systemic factors. Addressing these factors requires a holistic approach that includes patient education, improved communication, and tailored healthcare delivery strategies, especially for vulnerable populations in rural areas. Enhanced reminder systems and streamlined appointment management could serve as pivotal interventions to reduce non-attendance rates, ultimately improving healthcare outcomes and resource utilization.

## Introduction and background

Non-attendance at scheduled medical appointments, also commonly referred to as "no-shows," missed appointments, or "did not attends" is a prevalent issue in health care [1]. Each year, countless patients in various healthcare settings do not attend scheduled appointments [2]. The causes of missed appointments are not well understood. Perceptions of patients who miss appointments vary: some are perceived as vulnerable and understood to be living with multiple health issues and challenging life circumstances, while in other cases, they are viewed with irritation, as misusing valuable appointment slots that could benefit other patients in more urgent need.

The rate of missed appointments varies between 5% and 30%, depending upon the country, health care, and clinical environment [3-5]. For instance, in the United Kingdom (UK), the Health and Social Care Information Centre reported that between April 2011 and March 2012, 6.8 million or 7.5% of hospital outpatient appointments were not attended [6]. In the United States (US), Williams and Pepper (2009) reported that approximately 20% of physical therapy appointments were missed [7] whereas, in Ireland, Murphy and Taaffe (2019) noted that 16.67% of outpatient appointments were not kept in 2015 [8]. One hospital in Saudi Arabia, King Saud University Hospital, has a non-attendance rate of 24.8% [9].

In 2011, healthcare spending in the US reached 2.7 trillion US dollars (USD), amounting to 17.9% of its gross domestic product (GDP) and surpassing the combined healthcare expenditure of other countries [10]. Although there has been a deceleration in the rate of US healthcare spending growth due to economic downturns, projections suggest an upswing in tandem with recovery. For the last 20 years, the rate at which US healthcare expenses have grown has outpaced GDP growth [11]. This trend highlights the critical need for cost reduction in the healthcare sector. In 2012, the Institute of Medicine highlighted how inefficiencies in healthcare service delivery, including the issue of missed appointments, led to a substantial financial loss of 190 billion USD in the US healthcare system [12].

Missed appointments significantly undermine clinical outcomes. They interrupt the continuity of care, delay necessary treatments, and compromise the patient-doctor relationship, ultimately escalating healthcare costs [13]. When patients fail to attend clinical appointments, it detrimentally affects their care, depriving them of crucial treatment or screening opportunities [2]. In the context of diabetes management, non-attendance at appointments has been identified as a critical factor in suboptimal disease control. A 2000 US study found that neglecting to attend appointments was a predominant factor associated with treatment failure [14]. Furthermore, missed appointments prevent other patients from accessing timely care, increase operational strain on healthcare systems, and extend the booking and waiting times for others in the clinic [15]. Notably, such absences result in lost opportunities for earlier interventions for other patients, impacting the overall quality of care they receive [2]. In addition, healthcare professionals face increased stress and challenges in managing clinical loads due to patient non-attendance.

Despite the significance of the issue, comprehensive reviews focusing on the underlying causes of hospital appointment non-attendance are lacking. Addressing this research gap, this review aims to adopt a broad perspective on the factors contributing to non-attendance in hospital settings. The core research question guiding this review is: What are the various factors and reasons associated with patients' non-attendance at scheduled hospital appointments?

## Review

In the present narrative review, the exploration of missed hospital appointments incorporates the use of the terms "reasons" and "factors," distinguished by their usage in different study types. Quantitative research often utilizes the term "factors," whereas "reasons" are predominantly identified in qualitative research focusing on why appointments are missed. This review separates factors and reasons, providing a clearer understanding of each.

Factors influencing non-attendance

Several sociodemographic factors are highlighted as influential in non-attendance, including gender, age, marital status, education, and the geographical distance from the patient's home to the hospital. The source of the appointment request is also influential. These are regarded as tangible factors, as they are measurable and interpretable with relative ease.

Gender

The role of gender in influencing hospital appointment non-attendance has been scrutinized in 17 studies in diverse global contexts, including the US [16-18], UK [19,20], Saudi Arabia [1,21], Denmark [22], Nigeria [23,24], Australia [25-27], Singapore [28], Spain [29], Argentina [30], and Switzerland [31]. The scale of these studies ranges significantly, with sample sizes from 165 [25] to 113,716 [30] (Table 1). These investigations employed a range of statistical analyses, including the chi-square test [1,17,19-21,23,24,27,29,31], logistic regression [22,25,27-30], and linear regression [16] to explore the link between gender and non-attendance. Ten studies reported that gender has a significant role in predicting patient non-attendance [1,18-22,24,26-28].

**Table 1:** Characteristics of studies on the association between gender and non-attendance

Author (year)	Study setting	Country	Sample size	Study design/statistical analysis	Outcomes related to gender	References
Adams et al. (2004)	Department of Gastroenterology and Hepatology, Royal Perth Hospital	Australia	2157	Observational study (retrospective audit)/chi-square test	Non-attendance was significantly associated with the male gender with p = 0.001	[26]
Akinniyi & Olamide (2017)	Diabetic outpatients’ clinic, the University College Hospital	Nigeria	500	Observational study (cross-sectional)/chi-square test	Non-attendance was significantly associated with diabetic males (p = 0.004) and hypertensive females (p = 0.003)	[24]
Alhamad (2013)	General Clinic, Military Hospital	Saudi Arabia	760	Observational study (cross-sectional)/chi-square test	Non-attendance was significantly associated with the female gender with p = 0.001	[1]
AlRowaili et al. (2016)	Division of Magnetic Resonance Imaging, King Abdul-Aziz Medical City	Saudi Arabia	904	Observational study (cross-sectional)/chi-square test	Non-attendance was significantly associated with the female gender (p = 0.0001)	[21]
Blæhr et al. (2016)	Department of Radiology in Silkeborg Regional Hospital and the Department of Orthopedic Surgery in Viborg Regional Hospital	Denmark	86524	Observational study (cross-sectional) from June 2013 to March 2015/logistic regression analysis	Non-attendance was significantly associated with the male gender (p-value was not provided)	[22]
Bofill et al. (2011)	Immunology Clinic, Jackson Memorial Hospital	The United States of America	189	Observational study (cross-sectional)/multiple linear regression analysis	Non-attendance was not significantly associated with gender	[16]
Corfield et al. (2008)	Colorectal Surgical Clinic, St. Thomas’ Hospital	The United Kingdom	686	Observational study (prospective audit)/chi-square test	Non-attendance was significantly associated with males (p = 0.001)	[20]
Farley et al. (2003)	Cardiac Rehabilitation Unit, Queen Elizabeth Hospital	Australia	165	Observational study (prospective audit)/logistic regression analysis	Non-attendance was not significantly associated with gender (p = 0.11)	[25]
Giunta et al. (2013)	Outpatient clinics, the Italian Hospital of Buenos Aires	Argentina	113716	Observational study (retrospective cohort)/logistic regression analysis	Non-attendance was not significantly associated with gender	[30]
Hamilton et al. (2002)	Outpatient clinics, Royal Devon and Exeter Hospital	The United Kingdom	1972	Observational study (prospective cohort)/chi-square test	Non-attendance was significantly associated with males with p = 0.03	[19]
Harvey et al. (2017)	Radiology Department, Massachusetts General Hospital	The United States of America	54652	Observational study (retrospective cohort)/logistic regression analysis	Non-attendance was significantly associated with males (p < 0.001)	[18]
Lehmann et al. (2017)	General Internal Medicine Outpatient Clinic, the Geneva University Hospitals	Switzerland	1296	Observational study (retrospective cohort)/chi-square test	Non-attendance was not found to be significantly associated with gender	[31]
Low et al. (2016)	Diabetes Clinic, Khoo Teck Puat Hospital	Singapore	13244	Observational study (retrospective cohort)/logistic regression analysis	Non-attendance was found to be significantly associated with males (p = 0.013)	[28]
Mander et al. (2018)	Medical Imaging Department, Toowoomba Hospital	Australia	13458	Observational study (cross-sectional)/chi-square test and logistic regression analysis	Non-attendance was found to be significantly associated with males (p <0.001)	[27]
Mbada et al. (2013)	Physiotherapy Department, the Obafemi Awolowo University Teaching Hospitals	Nigeria	930	Observational study (retrospective audit)/chi-square test	Non-attendance was not found to be significantly associated with gender (p = 0.205)	[23]
Ootes et al. (2012)	Orthopaedic Clinic, Massachusetts General Hospital	The United States of America	665	Observational study (retrospective cohort)/chi-square test	Non-attendance was not found to be significantly associated with gender	[17]
Sola-Vera et al. (2008)	Gastroscopy Clinic, Hospital General Universitario (de Elche)	Spain	1807	Observational study (prospective cohort)/chi-square test and logistic regression analysis	Non-attendance was not found to be significantly associated with gender	[29]

Most studies investigating the correlation between gender and non-attendance at hospital appointments indicate that male patients are more prone to miss appointments. This pattern is notably prevalent in rural and local healthcare environments. Mander et al. (2018) suggest that this may be attributed to men's greater self-reliance and their reluctance or aversion to seeking help or medical assistance [27]. Nonetheless, one investigation found a link between gender and specific medical conditions, noting that men were more likely to skip appointments if they had diabetes, whereas women were more inclined to do so if they had hypertension [24]. Conversely, in two of the 10 studies where gender emerged as a significant factor, female patients exhibited a higher incidence of non-attendance than their male counterparts [21,26]. In the context of Saudi Arabia, cultural norms and legal restrictions, such as the prohibition against women driving, were cited as influential factors in these discrepancies [1,21], emphasizing the complexity of gender as a factor in non-attendance.

Seven studies did not establish a statistically significant relationship between gender and patient non-attendance [16,17,23,25,29-31]. Bofill et al. (2011), in a study focusing on critical cases involving the human immunodeficiency virus (HIV), proposed a rationale for their findings wherein the imperative for patients to be vigilant about their health may influence attendance [16]. However, the other six studies did not directly address the lack of a significant link between gender and non-attendance.

Research design, methods of data collection, and the size of the study population did not appear to affect the relationship between gender and non-attendance at hospital appointments. Employing various statistical analyses, such as the chi-square test, logistic regression, and linear regression, did not significantly impact the conclusions regarding the gender-non-attendance relationship, indicating that the observed variation was independent of the chosen statistical methodology.

Age

The statistical relationship between age and non-attendance at hospital appointments was analyzed in 17 international studies, conducted in the US [16-18], UK [19,32,33], Saudi Arabia [1], Denmark [22], Nigeria [23,24], Australia [26,27], New Zealand [34], Singapore [28], Argentina [30], and Switzerland [31]. These studies spanned various healthcare settings, including radiology [18,27,35], internal medicine [30,31], gynecology [32], endoscopy [26], general outpatient services [1,17,19], HIV/AIDS clinics [16], physiotherapy [23], diabetes care [24,28], orthopedics [22], rheumatology [34], and pain management.

These investigations, with sample sizes ranging from 189 [16] to 113,716 patients [30], scrutinized the potential link between patient age and non-attendance (Table 2). Various statistical methods were utilized across these studies, including the chi-square test [1,17,23,24,27,31,34], t-test [19,26,32,33,35], logistic regression [18,22,27,28,30,35], linear regression [16], Mann-Whitney test [34], and point biserial correlation test [17]. Age was analyzed as both a continuous and a categorical variable in these studies: some treated age continuously [16,19,26,32-35] while others considered it categorically [1,18,22-24,27,30,31,27]. Low et al. (2016) examined age as both a continuous (age) and a categorical variable (age group), offering a nuanced perspective on how age influences non-attendance [28].

**Table 2:** Characteristics of studies on the association between age and non-attendance

Author (year)	Study setting	Country	Sample size	Study design/statistical analysis	Outcomes related to age	References
Adams et al. (2004)	Department of Gastroenterology and Hepatology, Royal Perth Hospital	Australia	2157	Observational study (retrospective audit)/T-test	Non-attendance was significant among younger patients (p < 0.001)	[26]
Akinniyi & Olamide (2017)	Diabetic outpatients’ clinic, the University College Hospital	Nigeria	500	Observational study (cross-sectional)/chi-square test	Non-attendance was significantly associated with age (hypertension, p = 0.021; diabetes, p = 0.024)	[24]
Alhamad (2013)	General Clinic, Military Hospital	Saudi Arabia	760	Observational study (cross-sectional)/chi-square test	Non-attendance was significantly associated with young patients (age group = 0-20, p = 0.001)	[1]
Blæhr et al. (2016)	Department of Radiology in Silkeborg Regional Hospital and the Department of Orthopaedic Surgery in Viborg Regional Hospital	Denmark	86524	Observational study (cross-sectional) from June 2013 to March 2015/logistic regression analysis	Non-attendance was significantly associated with young patients (p < 0.01)	[22]
Bofill et al. (2011)	Immunology Clinic, Jackson Memorial Hospital	The United States of America	189	Observational study (cross-sectional)/multiple linear regression analysis	Non-attendance was significantly associated with young patients (p < 0.014)	[16]
Rogan et al. (2015)	Pain clinics, Birmingham City Hospital	The United Kingdom	3591	Observational study (retrospective cohort)/T-test	Non-attendance was significant among younger patients (p = <0.05)	[33]
Giunta et al. (2013)	Outpatient clinics, the Italian Hospital of Buenos Aires	Argentina	113716	Observational study (retrospective cohort)/logistic regression analysis	Non-attendance was significantly associated with age >65 years (p < 0.001)	[30]
Hamilton et al. (2002)	Outpatient clinics, Royal Devon and Exeter Hospital	The United Kingdom	1972	Observational study (prospective cohort)/T-test	Non-attendance was significant among younger patients (p < 0.001)	[19]
Harvey et al. (2017)	Radiology Department, Massachusetts General Hospital	The United States of America	54652	Observational study (retrospective cohort)/logistic regression analysis	Non-attendance was significantly associated with young patients (p = 0.05)	[18]
Lehmann et al. (2017)	General internal medicine outpatient clinic, the Geneva University Hospitals	Switzerland	1296	Observational study (retrospective cohort)/chi-square test	Non-attendance was significantly associated with young patients (p < 0.0014)	[31]
Low et al. (2016)	Diabetes Clinic, Khoo Teck Puat Hospital	Singapore	13244	Observational study (retrospective cohort)/logistic regression analysis	Non-attendance was significantly associated with young patients (p < 0.001)	[28]
Mander et al. (2018)	Medical Imaging Department, Toowoomba Hospital	Australia	13458	Observational study (cross-sectional)/chi-square test and logistic regression analysis	Non-attendance was not significantly associated with age, except 45-54 years (p < 0.001)	[27]
Mbada et al. (2013)	Physiotherapy Department, the Obafemi Awolowo University Teaching Hospitals	Nigeria	930	Observational study (retrospective audit)/chi-square test	Non-attendance was not significantly associated with the age (p = 0.216)	[23]
Milne et al. (2014)	Rheumatology outpatient clinics, Hutt Hospital	New Zealand	1953	Observational study (case-study)/Mann–Whitney non-parametric test	Non-attendance was significantly associated with young patients (p ≤ 0.001)	[34]
Ooi et al. (2021)	Radiology Department, Changi General Hospital	Singapore	59748	Observational study (retrospective audit)/chi-square, T-test, logistic regression analysis	Non-attendance was significantly associated with young patients (p < 0.001)	[35]
Ootes et al. (2012)	Orthopaedic Clinic, Massachusetts General Hospital	The United States of America	665	Observational study (retrospective cohort)/point biserial correlation test	Non-attendance was not significantly associated with the age	[17]
Pillai et al. (2012)	Gynecology outpatient clinic, North Middlesex University Hospital	The United Kingdom	6900	Retrospective study, the second arm a prospective case-control study/T-test	Non-attendance was significant among younger patients (p = 0.002)	[32]

The findings predominantly suggest a negative correlation between age and the probability of non-attendance at hospital appointments. It was consistently observed that younger patients are more likely to miss appointments in comparison with older patients [1,16,18,19,22,26,28,31-35]. Childcare responsibilities, work commitments, and better health status may explain the higher tendency among younger patients to miss appointments. A study of patients with HIV suggested that younger people might be more prone to missing appointments due to a shorter duration of living with the disease [16]. Younger patients, who may feel healthier and experience fewer symptoms, might perceive less necessity to adhere to their appointment schedules, potentially contributing to the elevated non-attendance rates among this group, as noted in the context of HIV [16]. While Akinniyi and Olamide (2017) identified age as a significant factor in non-attendance, the study did not differentiate between younger and older patients in terms of their likelihood of missing appointments [24]. Mander et al. (2018) highlighted the 45-54 years age group as significantly linked to non-attendance, the reasons for which were not clarified, while a significant correlation was not found for other age groups [27]. Interestingly, only Giunta et al. (2013) showed a significant pattern of non-attendance among older patients, specifically those aged over 65 years [30]. This study identified a trimodal distribution with peaks at ages 30-34, 65-69, and 80-84 years. However, the relevance of age was only noticeable in the univariate analysis and was not included in the final predictive model due to its insignificance, which could have compromised the model's accuracy.

Two studies [17,23] found no significant correlation between age and the likelihood of missing medical appointments. Mbada et al. (2013) observed a tendency for older individuals to miss appointments more frequently but this did not achieve statistical significance [23]. This pattern might be explained by a requirement among older patients to be accompanied to their appointments, for example, by a family member, as such, the absence of a companion on the scheduled day could lead to non-attendance. Additionally, obstacles related to transportation or health issues may have a role in this observed trend.

The choice of statistical analysis, whether chi-square test, t-test, binary logistic regression, or linear regression, did not alter the study outcomes, suggesting that the type of statistical method selected did not significantly impact the results obtained. Therefore, the association (or lack thereof) between age and non-attendance at appointments seemed consistent regardless of the statistical techniques/tools applied in the research.

Marital Status

Research exploring the influence of marital status on the likelihood of missing hospital appointments has been conducted in various contexts and countries, including the US [18,36-39], Saudi Arabia [1,21], the UK [32], Nigeria [40,41], Taiwan [42], and Thailand [43], with a broad range of sample sizes (Table 3). The smallest study by Adeponle et al. (2007) included 223 patients, whereas the largest by Peng et al. (2014) encompassed 881,933 patients [37,40]. The 12 studies included in the present review utilized the chi-square test [1,21,32,40], logistic regression [18,37,39,42,43], or a combination of the two statistical methods [36,38,41] to analyze how marital status correlates with patient non-attendance.

**Table 3:** Characteristics of studies on the association between marital status and non-attendance

Author (year)	Study setting	Country	Sample size	Study design/analysis test	Outcomes related to marital status	References
Adeponle et al. (2007)	Outpatient clinics, Federal Neuro-Psychiatric Hospital	Nigeria	223	Observational study (cross-sectional)/chi-square test	Non-attendance was not significantly associated with marital status	[40]
Akhigbe et al. (2014)	Outpatient clinics, regional psychiatric hospital	Nigeria	310	Observational study (prospective audit)/chi-square test and binary logistic regression analysis	Non-attendance was significantly associated with singles and patients who were living alone (p < 0.01)	[41]
Alhamad (2013)	General Clinic, Military Hospital	Saudi Arabia	760	Observational study (cross-sectional)/chi-square test	Non-attendance was not significantly associated with marital status (p = 0.52)	[1]
AlRowaili et al. (2016)	Division of Magnetic Resonance Imaging, King Abdul-Aziz Medical City.	Saudi Arabia	904	Observational study (cross-sectional)/chi-square test	Non-attendance was not significantly associated with marital status (p = 0.328)	[21]
Cheng et al. (2014)	Kaohsiung Municipal Kai-Syuan Mental Hospital	Taiwan	802	Observational study (cross-sectional)/logistic regression analysis	Non-attendance was not significantly associated with marital status (p = 0.986)	[42]
Daggy et al. (2010)	Outpatient Clinics, Midwestern Veterans Affairs hospital	The United States of America	5446	Retrospective study, the second arm a prospective case-control study/chi-square test and logistic regression analysis	Non-attendance was significantly associated with non-married (p < 0.0001)	[36]
Harvey et al. (2017)	Radiology Department, Massachusetts General Hospital	The United States of America	54652	Observational study (retrospective cohort)/logistic regression analysis	Non-attendance was significantly associated with marital status (p < 0.001)	[18]
Menendez & Ring (2015)	Orthopedic Clinic, Massachusetts General Hospital	The United States of America	14793	Observational study (retrospective cohort)/chi-square test and logistic regression analysis	Non-attendance was significantly associated with marital status (p < 0.001); single (p = 0.042) and widowed (p = 0.044)	[38]
Partin et al. (2016)	Colonoscopy unit, Veterans Affairs Hospital	The United States of America	27944	Observational study (cross-sectional)/logistic regression analysis	Non-attendance was significantly associated with non-married (p < 0.0001)	[39]
Peng et al. (2014)	Veterans Affairs Hospital (Level 1)	The United States of America	881933	Observational study (retrospective cohort)/logistic regression analysis	Non-attendance was significantly associated with non-married (p < 0.01)	[37]
Pillai et al. (2012)	Gynecology Outpatient Clinic, North Middlesex University Hospital	The United Kingdom	6900	Retrospective study, the second arm a prospective case-control study/chi-square test	Non-attendance was significantly associated with non-married and divorced (p = 0.014)	[32]
Thongsai (2015)	Diabetic Outpatient Clinic, Lerdsin Hospital	Thailand	442	Observational study (cross-sectional)/logistic regression analysis	Non-attendance was not significantly associated with marital status (p = 0.756)	[43]

Seven studies identified marital status as a crucial factor in patient non-attendance at hospital appointments [18,32,36-39,41]. These studies predominantly found that married individuals were less likely to miss appointments, suggesting that support from a spouse, financial stability, and a heightened sense of responsibility could contribute to the trend [18,36,37,39]. Other investigations within this group [32,38,41] indicated that individuals who were single, divorced, widowed, or living alone had a higher propensity to miss their appointments.

Interestingly, Akhigbe et al. (2014) found marital status to be significant according to the chi-square test but not in logistic regression analysis [41], while both Daggy et al. (2010) and Menendez and Ring (2015) reported marital status as a significant factor across statistical methods [36,38].

Five studies did not establish a significant link between marital status and appointment non-attendance [1,21,40,42,43]. The variation in outcomes may be attributed to differences in clinical settings, which require patients to maintain regular follow-ups. Alternatively, cultural and socioeconomic variables specific to each study’s location may have influenced these findings.

Education

The association between a patient's education level and their likelihood of missing hospital appointments was investigated in studies conducted in Nigeria [40,44], South Africa [45], the US [17,18], Saudi Arabia [1,21], New Zealand [46], and Taiwan [42] (Table 4). The sample sizes ranged from 223 [40] to 54,652 patients [18]. The methodologies utilized were the chi-square test [1,40,44,46] and logistic regression [18,42], with three studies employing both statistical methods [17,21,44].

**Table 4:** Characteristics of studies on the association between education and non-attendance

Author (year)	Study setting	Country	Sample size	Study design/analysis test	Outcomes related to education	References
Adeponle et al. (2007)	Outpatient clinics, Federal Neuro-Psychiatric Hospital	Nigeria	223	Observational study (cross-sectional)/chi-square test	Non-attendance was not significantly associated with educational level	[40]
Alhamad (2013)	General Clinic, Military Hospital	Saudi Arabia	760	Observational study (cross-sectional)/chi-square test	Non-attendance was significantly associated with educational level (no formal education; p = 0.01)	[1]
AlRowaili et al. (2016)	Division of Magnetic Resonance Imaging, King Abdul-Aziz Medical City	Saudi Arabia	904	Observational study (cross-sectional)/chi-square test/logistic regression analysis	Non-attendance was significantly associated with educational level (lack of education: chi-square, p = 0.019; logistic regression, p = 0.027)	[21]
Cheng et al. (2014)	Kaohsiung Municipal Kai-Syuan Mental Hospital	Taiwan	802	Observational study (cross-sectional)/logistic regression analysis	Non-attendance was not significantly associated with educational level	[42]
Harvey et al. (2017)	Radiology Department, Massachusetts General Hospital	The United States of America	54652	Observational study (retrospective cohort)/logistic regression analysis	Non-attendance was significantly associated with educational level (p = <0.001>)	[18]
Meer & Loock (2008)	ENT/Oncology clinics, Tygerberg Academic Hospital	South Africa	305	Observational study (prospective, descriptive series with controls)/not provided in the study	Non-attendance was not significantly associated with educational level	[45]
Ootes et al. (2012)	Orthopedic Clinic, Massachusetts General Hospital	The United States of America	665	Observational study (retrospective cohort)/chi-square test and logistic regression analysis	Non-attendance was significantly associated with educational level (p < 0.05)	[17]
Omokanye et al. (2021)	Outpatient clinics, Federal Hospital Bida	Nigeria	380	Observational study (cross-sectional)/chi-square test	Non-attendance was significantly associated with educational level (p = 0.005)	[44]
Ramm et al. (2012)	Internal Medicine Clinic, Green Lane Hospital	New Zealand	324	Observational study (cross-sectional)/chi-square test	Non-attendance was significantly associated with educational level (p < 0.05)	[46]

Synthesis of these studies indicates a significant association between a patient’s level of education and the likelihood of attending scheduled appointments [1,17,18,21,44,46]. Specifically, individuals with higher educational attainment appeared more likely to attend scheduled appointments.

It is plausible that patients with higher educational achievements may possess a deeper understanding of their health conditions and the benefits of treatment, leading to better adherence to their appointment schedules. AlRowaili et al. (2016) found that individuals with higher education levels were less prone to miss appointments, reporting a marked difference in non-attendance rates between patients with a high school education or lower and those with a bachelor's degree or higher. In the study, the likelihood of missing an appointment was increased threefold among patients with lower levels of education [21]. This observation aligns with the notion that better-educated patients might have a more comprehensive understanding of health, contributing to their commitment to keeping appointments. Omokanye et al. (2021) found that missed appointments were more common among patients from lower socioeconomic backgrounds, often lacking formal education and engaged in low-wage occupations [44]. Many patients who did not attend appointments were found to be without formal education, predominantly worked as artisans, were retired, or were traders, and typically lived in rural settings. This finding accentuates the need to consider socioeconomic elements that may lead to missed appointments, particularly in rural populations where access to healthcare services is constrained. Interestingly, this study noted that individuals with tertiary education were more likely to miss appointments than those with only primary education [44].

While six of the nine studies identified a significant link between education and appointment adherence [1,17,18,21,44,46], three studies [40,42,45] did not find such a relationship. Van der Meer and Loock (2008) found that non-attending patients had marginally lower levels of education than those who attended, but this difference was not statistically significant, suggesting education might not always be a pivotal factor in appointment adherence [45]. Education level was not identified as a significant factor in non-attendance in the studies of Adeponle et al. (2007) and Cheng et al. (2014) [40,42]. However, the possible reasons for these findings are not explicitly clear and warrant further exploration to understand how various cultural and contextual elements may influence this relationship in different settings.

The methodology, data collection techniques, and sample sizes across these studies did not appear to influence the observed correlation between educational level and non-attendance. Additionally, the use of various statistical methods, whether the chi-square test, logistic regression, or both, did not alter the overall conclusions regarding the influence of education level on non-attendance. This consistency suggests that the choice of statistical method did not significantly affect the results, pointing to an inherent variability in the relationship between a patient's education level and their likelihood of missing a hospital appointment.

Distance

Seven studies examined the influence of the distance between the patient’s home and the hospital on their likelihood of attending appointments [21,36-40,47]. These studies, conducted in diverse populations, varied significantly in sample size, ranging from 160 [47] to 881,933 patients [37] (Table 5). The methodologies employed were the chi-square test [21,40,47] and logistic regression analysis [37,39], with two studies [36,38] using both.

**Table 5:** Characteristics of studies on the association between distance from the hospital and non-attendance

Author (year)	Study setting	Country	Sample size	Study design/analysis test	Outcomes related to distance	References
Adeponle et al. (2007)	Outpatient clinics, Federal Neuro-Psychiatric Hospital	Nigeria	223	Observational study (cross-sectional)/chi-square test	Non-attendance was not significantly associated with distance	[40]
AlRowaili et al. (2016)	Division of Magnetic Resonance Imaging, King Abdul-Aziz Medical City	Saudi Arabia	904	Observational study (cross-sectional)/chi-square test	Non-attendance was significantly associated with distance (p = 0.002)	[21]
Bhise et al. (2016)	Colonoscopy Unit, Tertiary Hospital, Houston	The United States of America	160	Observational study (prospective cohort)/chi-square test	Non-attendance was significantly associated with distance (p < 0.01)	[47]
Daggy et al. (2010)	Outpatient clinics, Midwestern Veterans Affairs Hospital	The United States of America	5446	Retrospective study, the second arm a prospective case-control study/chi-square test and logistic regression analysis	Non-attendance was significantly associated with distance (p < 0.0001)	[36]
Menendez & Ring (2015)	Orthopedic Clinic, Massachusetts General Hospital	The United States of America	14793	Observational study (retrospective cohort)/chi-square test and logistic regression analysis	Non-attendance was significantly associated with distance (nearby) (chi-square, p = 0.047 and logistic regression, p = 0.0020)	[38]
Partin et al. (2016)	Colonoscopy Unit, Veterans Affairs Hospital	The United States of America	27944	Observational study (cross-sectional)/logistic regression analysis	Non-attendance was not significantly associated with distance	[39]
Peng et al. (2014)	Veterans Affairs Hospital (Level 1)	The United States of America	881933	Observation study (retrospective cohort)/logistic regression analysis	Non-attendance was significantly associated with distance in 2^nd^ year (p < 0.01)	[37]

Outcomes regarding the impact of distance on appointment attendance varied, with some studies indicating a significant link between greater distance and non-attendance [21,36-38,47], while others found no such relationship [39,40]. The significant findings indicate that logistical challenges, financial constraints, and difficulty in allocating time for the appointment due to other commitments were key aspects of appointments missed due to distance. Bhise et al. (2016) noted that patients with access to board and lodging facilities had reduced logistical barriers, especially when traveling over 100 miles to reach the hospital [47]. These insights highlight that the availability of transportation, financial means, and accommodation services can mitigate the effect of distance on appointment attendance. Peng et al. (2014) emphasized that the farther patients had to travel to the clinic, the more likely they were to miss their appointments, suggesting that the inconvenience of distance might outweigh the motivation to attend [37].

Menendez and Ring (2015) provided a contrasting perspective in their study conducted in Boston, US, where patients living closer to healthcare facilities were more prone to miss hospital appointments [38]. This may be due to perceptions of ease of rescheduling among local residents, who may be more likely to prioritize sudden, competing responsibilities, believing they can easily rebook due to their proximity to the hospital. This situation also hints at potential differences in the socioeconomic backgrounds of urban versus rural residents, suggesting a complex interplay of factors influencing appointment attendance based on distance.

While most of the studies analyzed in this review identified distance from the hospital as a critical factor influencing patient non-attendance, two studies did not find a consistent association [40,39]. Partin et al. (2016) identified a strong link between distance and the likelihood of appointment cancellations but not directly with non-attendance [39]. Adeponle et al.’s (2007) study was unique, as it took place in the sole conventional psychiatric facility in the area, which might have limited patients’ options, particularly for those living far from urban centers [40]. The differing results observed across the studies might also stem from various contextual elements, such as the geographic setting or the presence of alternative healthcare facilities, impacting the relationship between distance and patient non-attendance.

Source of Referral

The source of referral is the person or entity who directs the patient to the healthcare service. This may be their primary care physician, the emergency department, or the patient acting on their own initiative [23]. Seven studies conducted in Nigeria [23], the UK [19,32], Australia [26,27], Spain [29], and Saudi Arabia [1] investigated the link between referral sources and non-attendance. Sample sizes in these studies ranged from 760 [1] to 13,458 patients [27] (Table 6). The investigations utilized the chi-square test [1,23,26,32] and logistic regression analysis [19,27,29] to explore this association.

**Table 6:** Characteristics of studies on the association between source of referral and non-attendance

Author (year)	Study setting	Country	Sample size	Study design/analysis test	Outcomes related to source of referral	References
Adams et al. (2004)	Department of Gastroenterology and Hepatology, Royal Perth Hospital	Australia	2157	Observational study (retrospective audit)/chi-square test	Non-attendance was significantly associated with the emergency department as the source of referral (p = 0.007)	[26]
Alhamad (2013)	General Clinic, Military Hospital	Saudi Arabia	760	Observational study (cross-sectional)/chi-square test	Non-attendance was significantly associated with the source of referral (p-value was not provided)	[1]
Hamilton et al. (2002)	Outpatient clinics, Royal Devon and Exeter Hospital	The United Kingdom	1972	Observational study (prospective cohort)/logistic regression analysis	Non-attendance was significantly associated with the general practitioner being the high referrer as the source of referral (p = 0.05)	[19]
Mander et al. (2018)	Medical Imaging Department, Toowoomba Hospital	Australia	13458	Observational study (cross-sectional)/chi-square test and logistic regression analysis	Non-attendance was significantly associated with the source of referral (p = 0.006)	[27]
Mbada et al. (2013)	Physiotherapy Department, the Obafemi Awolowo University Teaching Hospitals	Nigeria	930	Observational study (retrospective audit)/chi-square test	Non-attendance was significantly associated with the source of referral (p = 0.001)	[23]
Pillai et al. (2012)	Gynaecology Outpatient Clinic, North Middlesex University Hospital	The United Kingdom	6900	Retrospective study, the second arm a prospective case-control study/chi-square test	Non-attendance was not significantly associated with the source of referral (p > 0.05)	[32]
Sola-Vera et al. (2008)	Gastroscopy Clinic, Hospital General Universitario (De Elche)	Spain	1807	Observational study (prospective cohort)/chi-square test and logistic regression analysis	Non-attendance was significantly associated with the general practitioner as the source of referral (p < 0.0001)	[29]

A significant correlation between referral source and patient non-attendance was found in six of the seven studies [1,19,23,26,27,29]. Sola-Vera et al. (2008) showed that patients referred by general practitioners were more likely to miss appointments compared to those referred by specialists, such as gastroenterologists, as non-attendance rates were notably different between these groups [29]. Likewise, Alhamad’s (2013) research in Saudi Arabia found that specialist-referred patients were more likely to attend their appointments [1]. This pattern suggests that the type of practitioner making the referral can significantly impact a patient’s adherence to scheduled appointments. These findings indicate that referrals from general practitioners have higher non-attendance rates than those from specialists, emphasizing the influence of the referral source on patients’ attendance behavior.

Mander et al. (2018) took a unique approach in their study to explore the link between the source of referral and patient non-attendance, focusing on the medical specialty of the referrer and including a new category for referrals from outside the hospital’s standard service region [27]. This methodological variation allowed the researchers a deeper understanding of how the referral source impacts patient non-attendance, with particular reference to whether patients referred from beyond the hospital’s usual service area had different non-attendance rates compared to those referred by local providers. Their results align with the broader literature, indicating a significant association between the referral source and non-attendance.

However, Pillai et al. (2012) found no significant correlation between referral sources and non-attendance [32]. Their approach involved comparing non-attending (cases) from specialty and general gynecology outpatient clinics with attending patients (controls), matched by referral reason. The control group was selected via a randomized computer-generated process to reflect the broader patient population accurately. The data were then uniformly gathered using a standardized form to ensure consistency.

Sample size and study setting did not conclusively impact the relationship between referral source and non-attendance. Notably, Pillai et al.’s method of control selection, using a computer-generated technique, differed from other studies and did not alter the study’s outcome [32]. Similarly, the use of statistical analyses, such as chi-square and logistic regression, across studies did not affect the results, suggesting that the choice of statistical method was not a critical factor in determining the relationship between referral source and patient non-attendance.

Reasons influencing non-attendance

In contrast with the more measurable factors previously described, reasons for non-attendance have been identified based on qualitative research incorporating patients’ perspectives. These reasons, often termed "soft factors" are subjective and reflect personal experiences and perceptions, making them harder to quantify but crucial for understanding patient behavior. They are generally categorized as patient-centered or hospital-specific.

Patient-Centered Reasons

Patient-centered reasons encompass issues directly related to the individual, such as forgetting the appointment or having a scheduling conflict that makes attending the appointment impractical. Studies conducted in the UK [33,48,49], Nigeria [24,44], Germany [50], Ireland, Argentina [51], the US [47], Spain [29], Australia [52], and the Sultanate of Oman [5] identified forgetfulness as a prevalent reason for not attending appointments. However, it is important to acknowledge the complexity of non-attendance, suggesting that forgetfulness may not be as straightforward as it initially appears. Poll et al. (2017) emphasized that labeling forgetfulness as a reason for missing appointments could oversimplify the issue [53]. Alhamad (2013) noted that some patients receive notification of their appointments too late to attend them, which could contribute to the perception of forgetfulness being more than just an oversight [1].

Within the studies, forgetfulness is frequently listed as one of the top three reasons for patient non-attendance at appointments [29,51]. One study based on self-reported data collected by telephone interview found forgetfulness was the leading cause of missed appointments, representing 44% of total non-attendance. A study in the Sultanate of Oman identified forgetfulness as the third most common reason for non-attendance [5], with transportation issues cited as the primary reason. This finding might reflect the unique social and cultural context of Oman and its influence on the ranking of reasons for missing appointments. The accuracy of these findings may be impacted by the data collection methods used. For example, Alawadhi et al. (2022) initially collected open-ended responses before categorizing them based on a pre-established list, while Omokanye et al. (2021) employed a semi-structured questionnaire, and Briatore et al. (2019) combined two distinct approaches, a structured questionnaire with an open-ended question to investigate non-attendance reasons [5,44,51].

Appointment timing was also a notable reason affecting non-attendance [1,5,24,33,49,51,54-56], with patients often citing conflicts with work or personal responsibilities. In discussion of how work commitments affect non-attendance, Alhamad (2013) noted that these may be the patient’s own commitments or those of a third party, necessary for transportation or accompaniment, particularly if the patient is female or older [1]. Guedes et al. (2014) described patients struggling to attend morning appointments, although the study did not account for employment status, which could influence availability [54]. Patients’ preferences for appointments at specific times or days were also reported, with one study [55] noting a preference to attend mental health appointments on Fridays. This highlights how appointment timing can significantly impact patient attendance at healthcare facilities.

Hospital-Specific Reasons

Hospital-specific reasons contributing to non-attendance were predominantly categorized into four main areas: lengthy waiting times, absence of reminders, appointment management issues, and the patient-physician relationship [24,47,49,54,56].

Extended waiting times refer to a prolonged interval from when an appointment is scheduled to the actual date of the appointment [24,47,49,54,56]. Numerous studies have highlighted that extended waiting times for hospital appointments pose a significant challenge within healthcare systems [24,47,49,54,56,57], with a public poll showing 75% of respondents prioritized the reduction of waiting times. The correlation between long waiting periods and increased patient non-attendance at appointments is well-documented [58].

Reports suggested that the longer the waiting time, the higher the chance a patient will forget their appointment, experience symptom improvement, or decide to seek care elsewhere [29]. Eades et al. (2019) reported that some participants attributed their non-attendance to the lengthy gaps between their appointments, leading them to forget to attend [49]. In Yang et al.'s (2020) study, patients indicated their non-attendance was due to protracted waiting times, prompting concerns about the progression of their illness and leading some to seek alternative treatment facilities [56]. The literature lacks a reliable definition of what constitutes a "long" waiting period, with some studies using the term to refer to the duration patients spend waiting at the clinic [48,47]. Patient dissatisfaction with outpatient care, influenced by long waiting times and perceived clinic disorganization, was also a substantial driver of decisions to miss appointments [48].

Some studies have reported the absence of reminders as a key driver of non-attendance [5,20,52,55,59]. For instance, Claveau et al. (2020) found that patients who did not attend their virtual appointments were often expecting a confirmation or reminder and that non-receipt was a primary reason for non-attendance [55]. Lyon and Reeves (2006) found that many patients were surprised or claimed they were awaiting a reminder in response to inquiries about their absence [59]. Corfield et al. (2008) noted that administrative errors leading to patients not receiving outpatient letters were often cited as reasons for missed appointments [20]. Research comparing a group receiving appointment reminders to a control group that received no reminder highlighted a significant difference in their attendance rates, strengthening the argument that the absence of reminders significantly contributes to non-attendance [60].

Additionally, the literature points to the complexities surrounding appointment management, including cancellation, rescheduling, or confirmation issues [20,61]. Corfield et al. (2008) reported that multiple patients attempted to contact hospitals to cancel unfeasible appointments but were unsuccessful due to a lack of response [20]. Survey respondents expressed frustration over not being able to communicate with hospital staff, leading some to personally visit the clinic to resolve their issues. This perceived communication breakdown could also discourage patients from attending future appointments [61]. The difficulty in reaching hospital staff to manage appointments was echoed by other participants who struggled to speak to staff on the telephone or leave messages [55]. Patients were also more likely to miss appointments when they perceived the appointment system to be flexible and negotiable, or when they believed staff may accommodate patients outside scheduled times.

Non-attendance at appointments was also attributed to patients' lack of awareness of the importance of keeping hospital appointments and an inadequate understanding of their health conditions [24,40,49,56,62]. In a Chinese study, patients' insufficient knowledge of their illnesses was noted as a reason for non-attendance, with patients often downplaying its severity if they noticed minor symptom improvements, leading them to consider appointments unnecessary [56]. Similarly, patients with diabetes minimized the impact of the condition on their daily activities, which contributed to missed appointments [49]. Lacking comprehension of the nature of their illness meant that patients did not fully grasp the potential risks or the severity of their conditions, especially when these did not disrupt their daily routines [49]. Another study highlighted that the asymptomatic nature of hypertension might lead to a lack of disease perception, particularly among patients under the age of 50 years, contributing to appointment non-attendance [62]. In other cases, patients' absence was linked to their unawareness of the benefits of regular medical consultations [24,40].

The doctor-patient relationship was an influential factor in appointment attendance in four studies [43,48,56,63]. Murdock et al. (2002) found that a small percentage of patients (3%) skipped their appointments due to concerns about being seen by a junior doctor, a reason not prevalent in other studies in this review [63]. The patient demographic, predominantly aged around 50 years and attending a gastrointestinal clinic, may have influenced their preference to be seen by a consultant. Yang et al. (2020) suggested that initial consultations in private clinics might not provide sufficient information or clarification of the patient's condition, leading them to doubt the doctor's intent and focus on their well-being. This skepticism made attending appointments challenging due to a trust shortfall in the doctor-patient interaction [56]. Abdulkadir et al. (2019) identified language barriers as creating challenges in the doctor-patient relationship, affecting appointment adherence, as patients lacked proficiency in the local language (Danish) and depended on translators [43]. A further study reported patients expressed discomfort with seeing a different doctor at each visit, which hindered the development of a meaningful doctor-patient connection [48].

## Conclusions

This narrative review explored various factors, such as age, gender, marital status, level of education, distance to the hospital, and referral source, which are correlated with non-attendance at medical appointments in the existing literature. Research conducted in different countries, utilizing various study designs, sample sizes, and statistical methods, was reviewed to assess how these six factors relate to non-attendance. It was found that, generally, younger patients are more prone to missing appointments, potentially due to external commitments or better health status. Gender was identified as a significant factor in non-attendance, with male patients more likely to miss appointments, especially in rural healthcare settings. Marital status emerged as an influential factor, with studies indicating that married patients are less likely to miss appointments, possibly due to the social support and financial stability marriage may provide. The source of the referral also affects non-attendance rates, with findings suggesting that patients referred by general practitioners show a greater tendency to miss appointments than those referred by specialists. Distance from the hospital had a variable impact on attendance, influenced by factors such as transportation challenges, financial issues, and competing personal obligations. Moreover, higher educational levels were correlated with lower non-attendance rates, demonstrating a link between education and patient adherence to scheduled healthcare appointments.

Reasons for non-attendance, as determined in qualitative research, were categorized into two main types: patient-centered and hospital-specific. Among patient-centered reasons, forgetfulness was frequently highlighted in various international studies as a primary reason for missing appointments, although the complexity of non-attendance suggests that other social and cultural factors may also be influential. Conflicts between the patients' schedules and their appointment times, as well as preferences for specific appointment times or days, were also identified as impacting attendance rates. Hospital-specific factors, such as long waiting periods, lack of reminders, inflexible appointment management, poor communication, and the quality of the patient-physician relationship were identified as contributing to non-attendance, causing patients to forget or skip their appointments. A lack of awareness among patients about the importance of appointment attendance and dissatisfaction with clinic organization were also noted as reasons for non-attendance. Addressing these patient-centered and hospital-specific issues is crucial for healthcare providers aiming to decrease non-attendance rates.
